# THz graphene-integrated metasurface for electrically reconfigurable polarization conversion

**DOI:** 10.1515/nanoph-2023-0916

**Published:** 2024-03-18

**Authors:** Li-Zhao Song, Andrew Squires, Timothy van der Laan, Jia Du

**Affiliations:** 2221 Commonwealth Scientific and Industrial Research Organisation (CSIRO), Lindfield, NSW, Australia

**Keywords:** electrical modulation, graphene, metasurface, polarization conversion, terahertz

## Abstract

Terahertz (THz) waves have been widely hailed as a key enabling technology for future sixth generation (6G) wireless networks. Dynamic modulation of their polarization states is of great attraction for high-capacity communications and anisotropic sensing. The development of such technology is, however, still in very early stage owing to the difficulties of realizing electrical reconfigurability for THz devices. Artificially constructed metasurfaces and new nanomaterials, such as graphene, have been shown to provide revolutionary platforms for manipulating and controlling the wave properties, especially at THz frequencies. This work leverages the light–matter interaction in a graphene-integrated metasurface functioning as an electrically reconfigurable THz polarization converter. A novel graphene-gold bilayer topology is applied to construct such a metasurface which enables wide-range electrical tunability of the polarization conversion. Under a *y*-polarized illumination, the reflected components of *x*- and *y*-polarizations are tuned dynamically through an external bias voltage across the metasurface, thereby producing an elliptically polarized wave with tuneable ellipticity and angle. By changing the voltage from 0 V to 12 V, the reflected polarization ellipticity has been tuned from −0.94 to −0.5 at around 240 GHz, featuring linear-to-circular and linear-to-elliptical polarization conversions. Meanwhile, the polarization angle has been modulated from 12° to −23° at around 236 GHz. This work provides an experimentally validated THz graphene-integrated metasurface with wide polarization modulation depths, low biasing voltages and simple configuration. It promises great potential for applications in future THz communications and sensing.

## 1 Introduction

The last decade has witnessed a rapid growth in terahertz (THz) technologies by exploiting the spectrum of 0.1–10 THz [1]. This is due to their great potential for high-data-rate communications [2] and high-resolution, non-invasive sensing [3]. To support various functions and applications, THz waves can be manipulated specifically in terms of the amplitude, phase, or polarization. Among them, polarization modulation is particularly attractive to communications for increasing channel capacity by enabling data transmissions in multiple polarization states [4]. Meanwhile, dynamic polarization control will facilitate chiral material characterizations and biomolecule probing [5], [6], [7], thus bringing substantial benefits to biology and analytical chemistry. To date, different methods have been proposed to enable polarization tunability by employing spintronic emitters [8], birefringent crystals [9], photoconductive antennas [10], and microelectromechanical systems (MEMS) [11], to name a few. Despite the effective polarization modulation, these traditional methods still face problems of either low modulation speed or limited applications for compact systems, required for THz applications.

On the other hand, metasurfaces, with two-dimensional (2-D) periodic unit cells, serve as a compact candidate with high flexibility for manipulating EM wave properties [12]. Many dynamic polarization converters based on metasurfaces/metamaterials have been reported at optical frequencies using various tuning mechanisms [13], [14], [15], [16]. Electrically reconfigurable metasurfaces are particularly useful to support high-speed dynamic wave control [17]. However, research in this area at the THz band is still in its infancy due to the lack of cost-efficient and high-performance electronic components to enable active tuning at THz frequencies [18]. To fill this research gap, new electrically controllable materials and devices are being developed, such as liquid crystals (LC) [19], vanadium dioxide (VO2) [20], and graphene nanomaterials [21], etc. Graphene, a 2-D layer of carbon atoms, possesses appealing electrical properties including a strong light–matter interaction, continuously electrically tunable conductivity, and ultrafast carrier dynamics [22], [23], [24], [25]. Graphene-integrated 2-D metasurfaces [21], [22] have been demonstrated to provide a wide carrier concentration tunability and a fast reconfiguration speed affected from its short carrier momentum relaxation time.

To date, several THz graphene-integrated metasurfaces have been investigated to enable reconfigurable polarization conversions in open literature [26], [27], [28], [29], [30], [31], [32], [33]. However, most of these are theoretical designs only. Very few works have been demonstrated with practical implementations and experimental verifications [34], [35], [36], [37]. Challenges lie in the great difficulty of fabricating graphene materials into complicated micro and nano structures with good reproducibility and parameter-controllability as well as achieving a high degree of electrical tunability with low biasing voltages. In Ref. [34], a metasurface, consisting of bright and dark metal resonators, was developed with applying an array of graphene patches at the gap of the dark resonators for active polarization modulation. By applying biasing voltages from −125 V to +25 V, the transmitted polarization angle has been electrically tuned within a range of 20° at 1.75 THz. In Ref. [35], an unpatterned graphene film was added under a metasurface that has dual chiral metallic layers. By changing the external biasing voltage from −35 V to +100 V, an electrical polarization control has been achieved with a tunable ellipticity of 0.55–0.98 and a greater than 20° angle tuning at around 2 THz. It is noticed that both the above-mentioned designs have unpatterned or simple patch-patterned graphene and require a very high biasing voltage (over 100 V) to achieve the mentioned polarization modulations, increasing system power consumptions and the risk of damaging the graphene film. On the other hand, ion gels have been employed for graphene-integrated devices to increase the polarization modulation depth with smaller biasing voltages [36], [37], [38]. However, charge response in the ion gel is slow, thus sacrificing the modulation speed.

In this work, we present a theoretical design and experimental verification of a reconfigurable THz polarization converter utilising a graphene-gold bilayer metasurface where graphene itself is patterned into the entire complex metasurface structure. Efficient electrical tuning is achieved with relatively small biasing voltages and without resorting to additional ion gel layers. This graphene-gold bilayer approach has addressed the lack of tunability of gold metasurfaces and poor resonant performance of graphene metasurfaces due to its high intrinsic loss.

## 2 Results and discussion

### 2.1 Design methodology and theory

**Figure 1:**
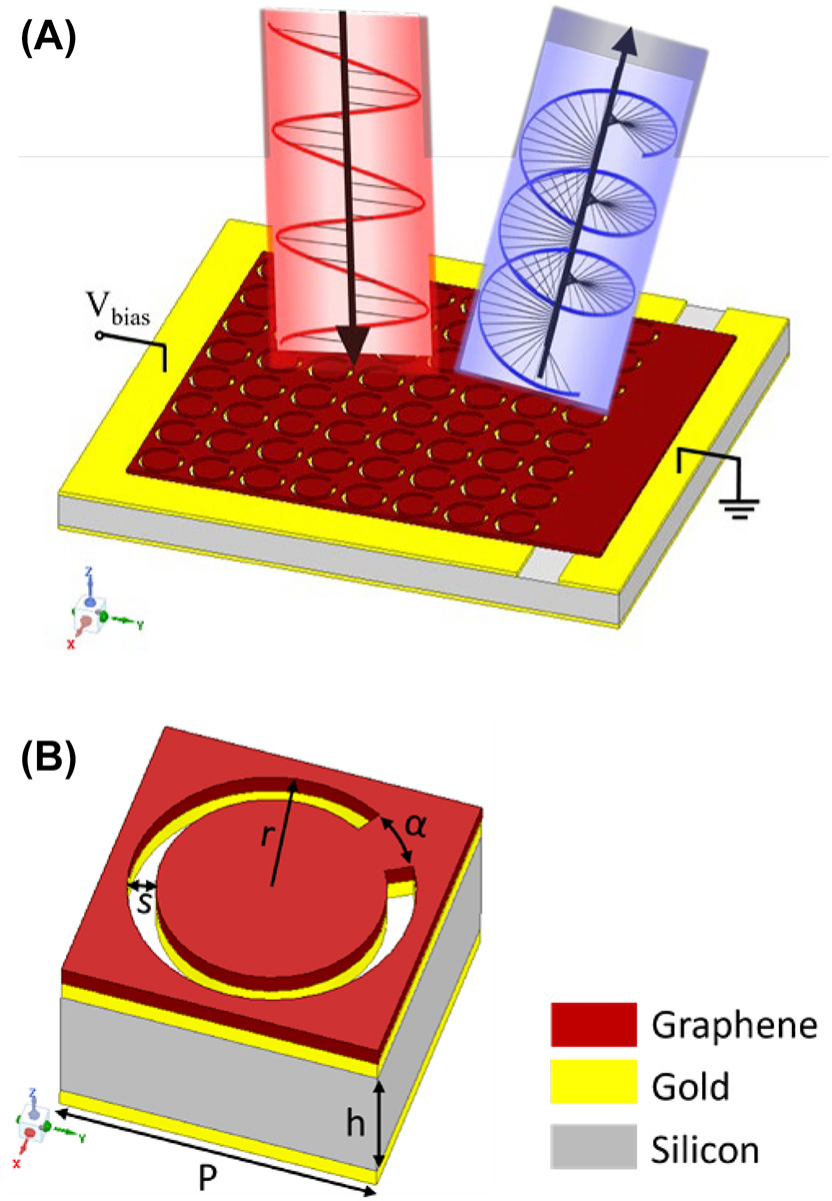
Schematics of the proposed structure. (A) Graphene integrated bilayer polarization converter, and (B) unit cell.

A schematic of the proposed graphene-integrated metasurface and its unit cell model are shown in Figure 1(A) and (B), respectively. A 0.285 mm thick silicon is selected as the substrate with a gold thin film on the bottom as the ground layer. On top of the substrate, a bilayer graphene-gold meta-structure with an array of periodically slotted split rings is designed for polarization conversion. The conductivity of the graphene layer is electrically tuned through a biasing voltage as illustrated in Figure 1(A). The unit cell has a periodicity of *P* = 0.23 mm. The slotted ring on the structure has a split angle of *α* = 30° and an outer radius of *r* = 0.1 mm with a slot width of *s* = 0.02 mm. The split is conducted at one corner along the diagonal of the lattice, thus producing an asymmetric unit-cell geometry and supporting anisotropic properties. This way, polarization conversion capability can be obtained. Meanwhile, the bilayer meta-structure, of which the graphene and gold layers have the same pattern, promises the exploitation of graphene tunability without destroying the desired polarization conversion function by the gold layer.

**Figure 2:**
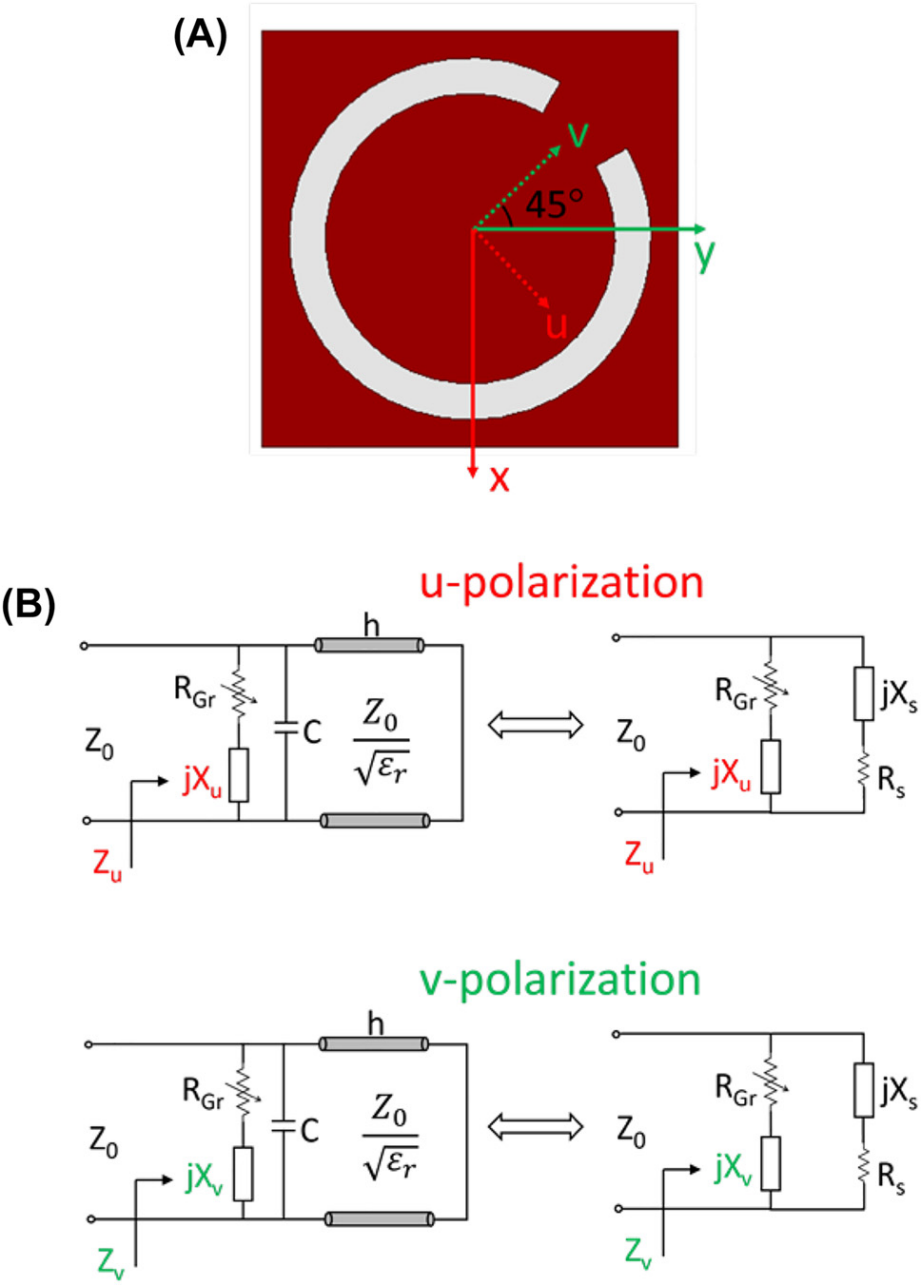
Unit cell illustration and circuit. (A) Structure top view. (B) Equivalent circuits for u- and v- polarizations.

A top view of the unit cell is shown in Figure 2(A). To analyze the polarization conversion mechanism, the original *x*–*y* coordinate is rotated by +45° to obtain a *u*–*v* coordinate as in Figure 2(A). Along *u*- and *v*-axes, the unit cell exhibits anisotropic properties due to their distinct physical geometries. Their equivalent circuits are provided in Figure 2(B), where *X*_*u*_ and *X*_*v*_ represent the reactance from inductive and capacitive effects of the slotted unit cell along *u*- and *v*-directions, respectively, *R*_Gr_ denotes the tunable resistance of the graphene film under different chemical potentials, *C* is the coupling capacitance between the top and bottom layers of the substrate, *Z*_0_ is the wave impedance in free space. The substrate is represented by an equivalent transmission line with a characteristic impedance of $Z_{0}/\sqrt{\varepsilon_{r}}$ , where *ε*_*r*_ is the dielectric constant of the silicon substrate as 11.9. The final equivalent circuits are shown in the right column of Figure 2(B), where *X*_*s*_ is utilized to represent the reactance from the transmission line and the coupling capacitance, *R*_*s*_ accounts for the substrate loss. Consequently, the reflection coefficients along *u*- and *v*-axes can be calculated with

(1a) $$\Gamma_{u,v}=\frac{Z_{u,v}-Z_{0}}{Z_{u,v}+Z_{0}},$$

(1b) $$Z_{u,v}=\frac{\left(R_{\text{Gr}}+jX_{u,v}\right)\left(R_{s}+jX_{s}\right)}{R_{\text{Gr}}+R_{s}+j\left(X_{s}+X_{u,v}\right)}.$$

Considering the unit cell illuminated by a *y*-polarized incident wave, it can be decomposed into *u*–*v* components as

(2) $$E_{i}=\hat{y}E_{0}=\frac{\sqrt{2}}{2}\left(\hat{u}E_{0}+\hat{v}E_{0}\right).$$

The reflected wave ***E***_*r*_ will be obtained as

(3) $$E_{r}=\frac{\sqrt{2}}{2}\left(\hat{u}E_{0}\Gamma_{u}+\hat{v}E_{0}\Gamma_{v}\right).$$

This can be expressed in *x*–*y* coordinate as

(4) $$E_{r}=\hat{x}E_{0}\left(\frac{\Gamma_{u}-\Gamma_{v}}{2}\right)+\hat{y}E_{0}\left(\frac{\Gamma_{u}+\Gamma_{v}}{2}\right)=\hat{x}E_{x}+\hat{y}E_{y}.$$

From (1a) and (1b), it is noticed that both Γ_*u*_ and Γ_*v*_ will be varied due to the tunable graphene resistance *R*_Gr_. Consequently, the reflected wave from (4) in terms of *x*- and *y*-components will feature different amplitudes and phases, thus enabling elliptical polarizations with tunable ellipticity *ϵ* and angle *θ*, which can be calculated from

(5) $$\epsilon =\frac{\left|E_{y}-jE_{x}\right|-\left|E_{y}+jE_{x}\right|}{\left|E_{y}-jE_{x}\right|+\left|E_{y}+jE_{x}\right|},$$

(6) $$\theta =\frac{\arg\left(E_{y}+jE_{x}\right)-\arg\left(E_{y}-jE_{x}\right)}{2}.$$

If $\left|∠\Gamma_{u}-∠\Gamma_{v}\right|$ = 180°, following (4), the *y*-polarized incident wave will be maximally converted into an *x*-polarized wave. The polarization conversion rate (PCR) is defined as

(7) $$\text{PCR}=\frac{|E_{x}{|}^{2}}{|E_{x}{|}^{2}+|E_{y}{|}^{2}}.$$

### 2.2 Modelling and simulation

To visualise the above explained unit cell functions, full-wave simulations have been conducted using the frequency domain solver in CST Studio Suite 2022. The silicon substrate has an electrical conductivity of 2.5 × 10^−4^ S/m. The gold film has an electrical conductivity of 4.561 × 10^7^ S/m. The complex conductivity of graphene is modelled using the intra-band term of the Kubo formula as

(8) $$\sigma \left(f\right)=-j\frac{e^{2}k_{B}T}{\pi \hbar^{2}\left(2\pi f-j\tau^{-1}\right)}\ln\left(2\left(1+\cosh\left(\frac{\mu_{c}}{k_{B}T}\right)\right)\right),$$

where *f* is the investigated THz frequency, *e* is the electron charge, *k*_*B*_ represents Boltzmann’s constant, *T* denotes the room temperature, *ℏ* is the reduced Planck’s constant, *τ* is the scattering time for charge carriers in graphene, *µ*_*c*_ is chemical potential.

**Figure 3:**
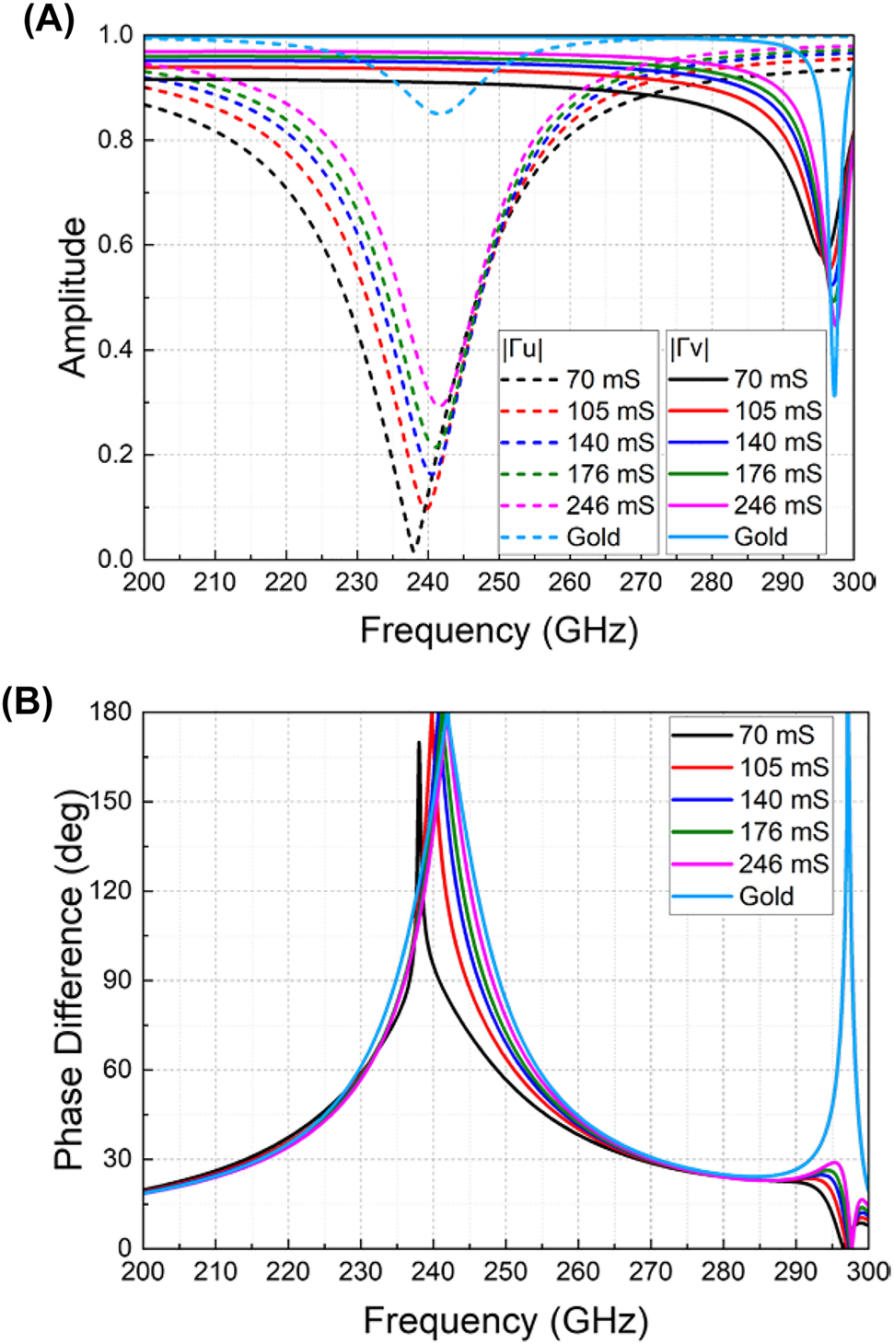
Simulated reflection coefficients under *u*- and *v*-polarized incidences versus different graphene conductivities. (A) Reflection amplitudes. (B) Reflection phase differences.

In simulation, unit cell boundaries are applied along both *x*- and *y*-axes with open boundaries assigned along *z*-axis. The unit cell is first characterized under *u*- and *v*-polarizations with different graphene conductivities in comparison to the unit cell with only gold structure. The simulated reflection coefficients in terms of their amplitudes (i.e., |Γ_*u*_| and |Γ_*v*_|) and phase differences (i.e., |∠Γ_*u*_–∠Γ_*v*_|) are illustrated in Figure 3(A) and (B). It is observed that the phase difference remains relatively stable as 180° at around 240 GHz for different conductivities, thereby promising the highest PCR around this frequency based on (4). The reflection amplitudes of the *u*- and *v*-polarizations vary distinctively at 240 GHz for different conductivities, thus enabling variable peak PCR values. Note that the operating frequency of 240 GHz is chosen for the potential application in our THz communication system under development at 240 GHz.

**Figure 4:**
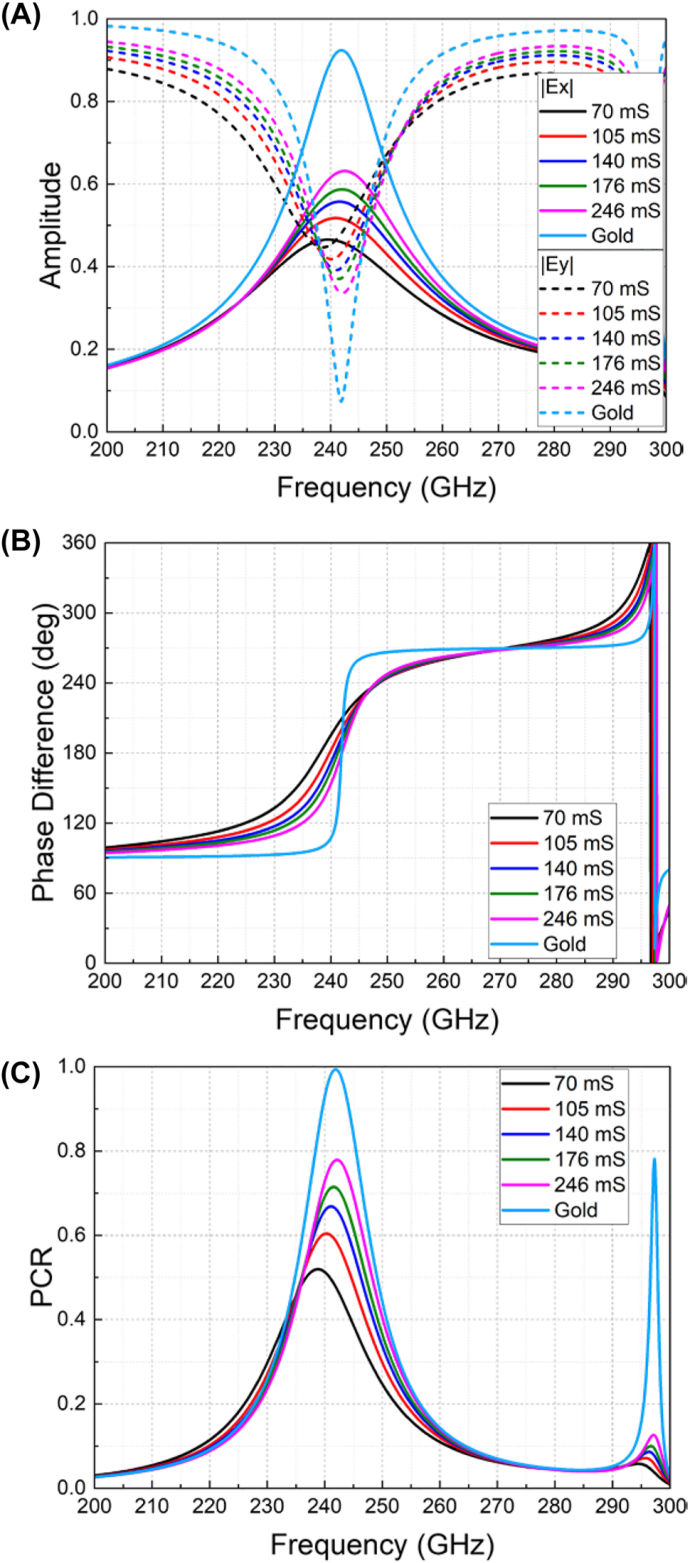
Simulated parameters under *y*-polarized incidence for different graphene conductivities. (A) Reflected amplitudes of *x*- and *y*-components. (B) Phase difference between the reflected *x*- and *y*-components. (C) PCR from *y*-polarized incidence to *x*-polarized outgoing wave.

**Figure 5:**
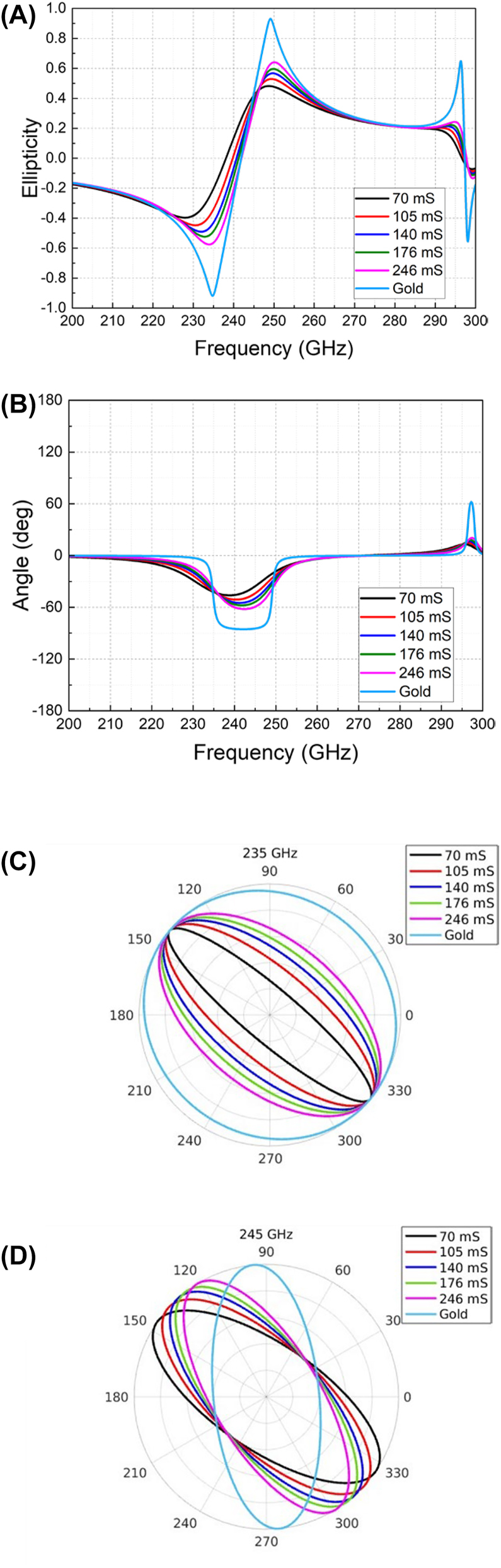
Simulated parameters under *y*-polarized incidence for different graphene conductivities. (A) Calculated ellipticity of the reflected polarization. (B) Rotation angle of the reflected polarization. (C) Polar plot of reflection polarizations at 235 GHz. (D) Polar plot of reflection polarizations at 245 GHz.

To verify the polarization conversion performance, the unit cell is then illuminated by a *y*-polarized incident wave, and its reflected components of *x*- and *y*-polarizations in terms of their amplitudes and phase differences are characterized in Figure 4(A) and (B) for different graphene conductivities. In Figure 4(A), intrinsic resonance of gold metasurface at 242 GHz is produced by the slotted split ring pattern due to the inductive and capacitive effects as illustrated in Figure 2(B). Since the graphene film mostly contributes to tunable resistance (*R*_Gr_ in Figure 2(B)) and the proposed bilayer metasurface has the same slotted pattern on the gold and graphene layers, the resonant frequencies with and without graphene layers change little. The resonant response amplitude has decreased in the bilayer structure due to the higher resistance of the graphene layer. The calculated PCRs are summarized in Figure 4(C). The peak PCR occurs at around 240 GHz for all different conductivities, coinciding with the conclusion drawn from the previous Γ_*u*_ and Γ_*v*_ characterizations. Meanwhile, the peak values of PCR are varied from 0.52 to 0.99. On the other hand, the variations of the amplitudes and phases of the *x*- and *y*-components will contribute to the active tuning of the reflection polarization state. Specifically, the polarization ellipticity and angle have been changed as given in Figure 5(A) and (B), respectively. Noticeably, at 235 GHz, the reflected polarization angle is stable as −40°, while its ellipticity has been varied from −0.92 to −0.2. At 245 GHz, the polarization ellipticity has a stable value of around 0.4, while the polarization angle has been changed from −85° to −35°. The negative values of the ellipticity denote left-handed polarizations, while the positive ones refer to right-handed polarizations. To visualize the polarization variations, polar plots of the reflected polarizations at 235 GHz and 245 GHz are given in Figure 5(C) and (D). It is noticed that a circular polarization has been obtained for pure gold structure at 235 GHz, which is evidenced by the equal amplitudes and 90° phase difference of *x*- and *y*-components from Figure 4(A) and (B), respectively.

### 2.3 Physical mechanism

**Figure 6:**
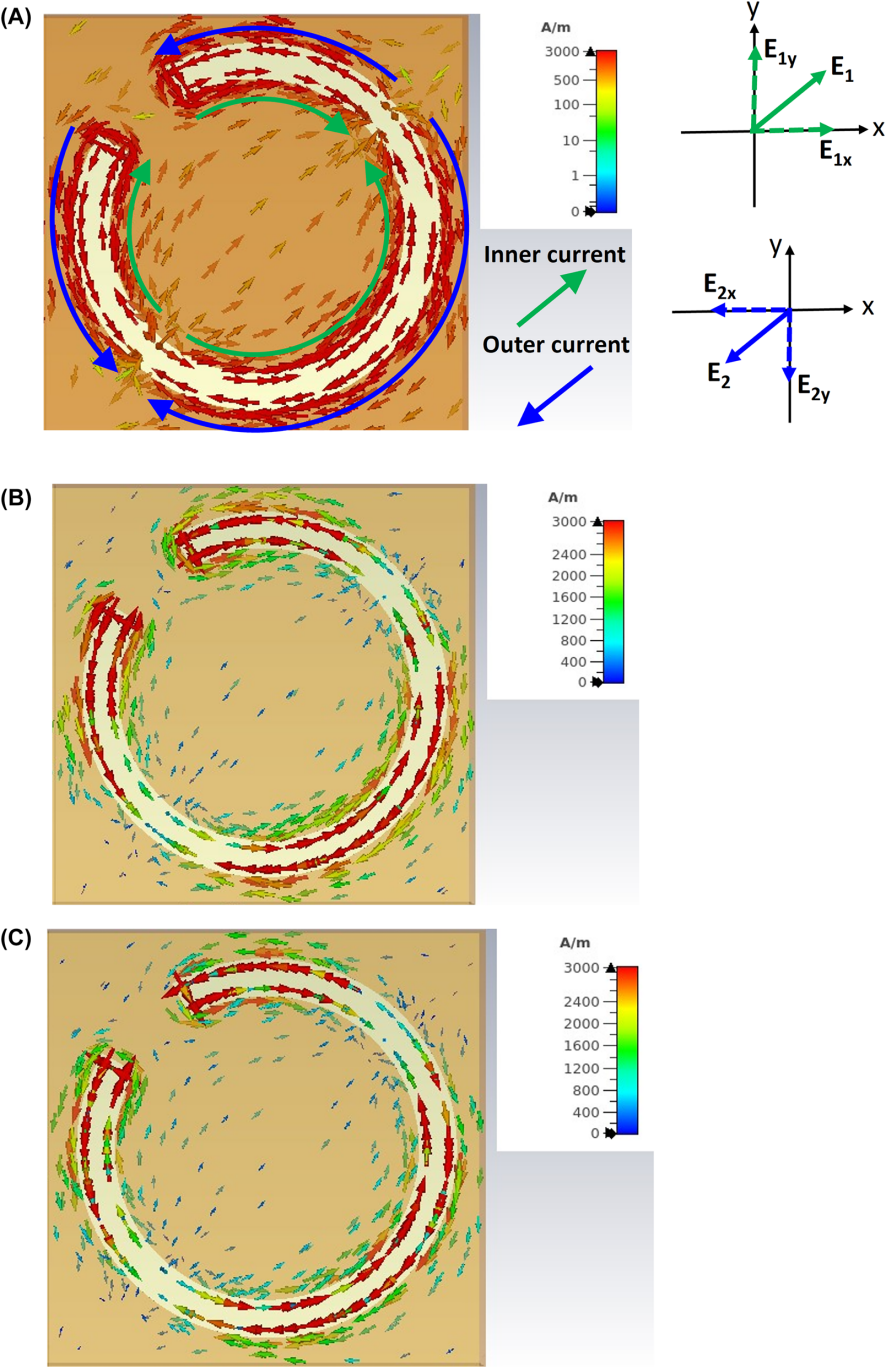
Surface current distributions on the bilayer structure at 240 GHz. (A) Pure gold unit model. (B) Model with 140 mS graphene conductivity. (C) Model with 70 mS graphene conductivity.

To investigate the physical mechanism of the proposed unit cell, the surface currents on the bilayer structure under *y*-polarized incidence have been analyzed for different conductivities at 240 GHz. Figure 6(A) shows the simulated current distribution of the pure gold unit model, where the currents along inner and outer peripheries of the ring slot produce electric fields of *E*_1_ and *E*_2_, respectively. Both can be decomposed into two components along *x*- and *y*-axes. The components of *E*_1*x*_ and *E*_2*x*_ will contribute to generating *x*-polarized reflection waves, thus enabling the polarization conversion from the *y*-polarized incidence into *x*-polarized radiation. Besides, the surface current distributions of the bilayer unit model with graphene conductivities of 140 mS and 70 mS are shown in Figure 6(B) and (C), respectively. It is noticed that the induced current directions on the bilayer structure are the same as the counterparts of the pure gold model. However, the current intensity has been dampened differently on the graphene-gold bilayer structure. As the graphene conductivity drops, the current intensity has decreased, leading to lower polarization conversion capabilities (i.e., smaller PCR values). This agrees with the results given in Figure 4(C).

### 2.4 Fabrication and experiment

**Figure 7:**
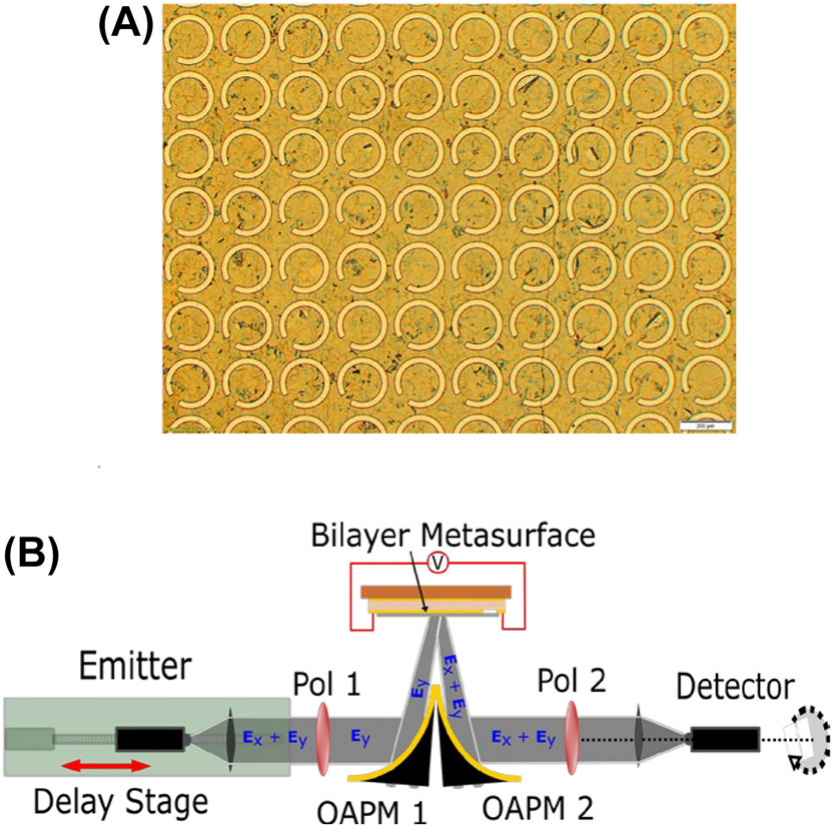
Experimental implementation. (A) Image of the fabricated graphene-integrated metasurface. (B) Measurement setup.

The experimental device was constructed on a 285 µm undoped float zone silicon substrate. An initial 220 nm gold layer was deposited using a DC sputtering technique on the back side as an RF ground layer (yellow bottom layer in Figure 1(A) and (B)). On the top side, a similar 220 nm gold layer sputtered with a hard mask to define the metasurface regions and contacts (yellow layer above the silicon substrate in Figure 1(A) and (B)). An in-house grown chemical vapor deposited (CVD) graphene film [39] was wet transferred onto the top side gold ensuring coverage of the contacts and metasurface regions. Using a similar approach developed in house and described in our previous work [21], the above slot-array design was transposed onto the graphene-gold using a photolithography process followed by O_2_ plasma etching to remove the graphene and Ar reactive ion etching to remove the gold. A final O_2_ plasma was used to clean the device chip. The fabrication process resulted in a well-defined and reproducible slotted metasurface array as seen in Figure 7(A).

As a proof of concept, the polarization converter was measured using THz time domain spectroscopy (THz-TDS) in a reflection geometry. A schematic of the setup used is shown in Figure 7(B). THz generation and detection were performed using two photoconductive antennas optically excited by a femtosecond laser source. The emitter antenna produces elliptically polarized light with the strong axis in-plane with the measurement benchtop. As such, a wire grid polarizer (Pol 1) was incident on the emitter arm to transmit this polarization, which we denote *Ey*, and remove other residual polarizations. The THz beam is then focused onto the bilayer metasurface and collected through two off axis paraboloid mirrors (OAPM). A second polarizer (Pol 2) is placed on the detector arm of the THz beam which can be rotated by 90° to probe the *Ex* and *Ey* component outputs from the device. The detector antenna is also mounted in a rotation stage to allow synchronisation of the detector and second polarizer. This minimizes the impact from any local polarization dependence of the detector.

For device measurement, a reference spectrum was obtained from a gold reflective surface positioned at the location of the bilayer metasurface with both polarizers positioned to permit the transmission of *y*-polarized waves. Each sample measurement was subsequently ratioed with this reference. Biasing voltages were applied in 3 V increments up to 12 V. Voltages higher than 12 V were not performed due to potential breakdown and irreparable damage to the graphene in the bilayer.

**Figure 8:**
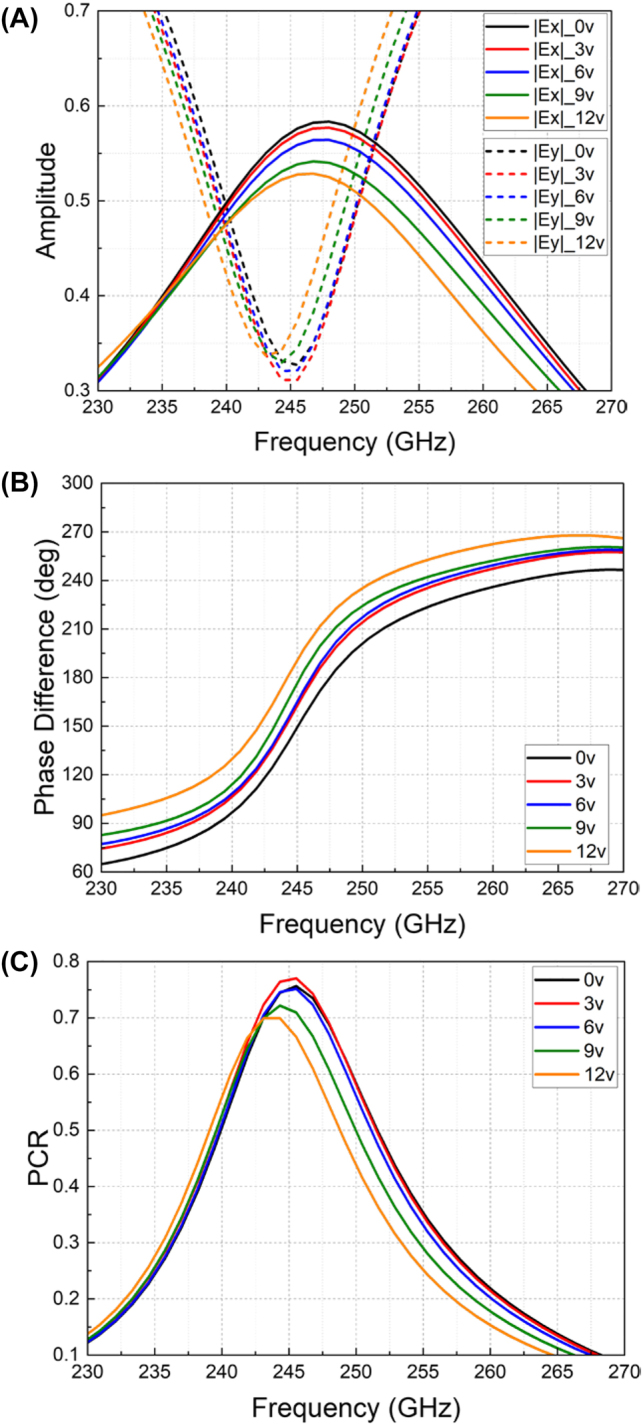
Measurement results. (A) Reflection amplitudes of *x*- and *y*-polarizations. (B) Phase differences of the reflected *x*- and *y*-components. (C) PCR variations under different biasing voltages.

**Figure 9:**
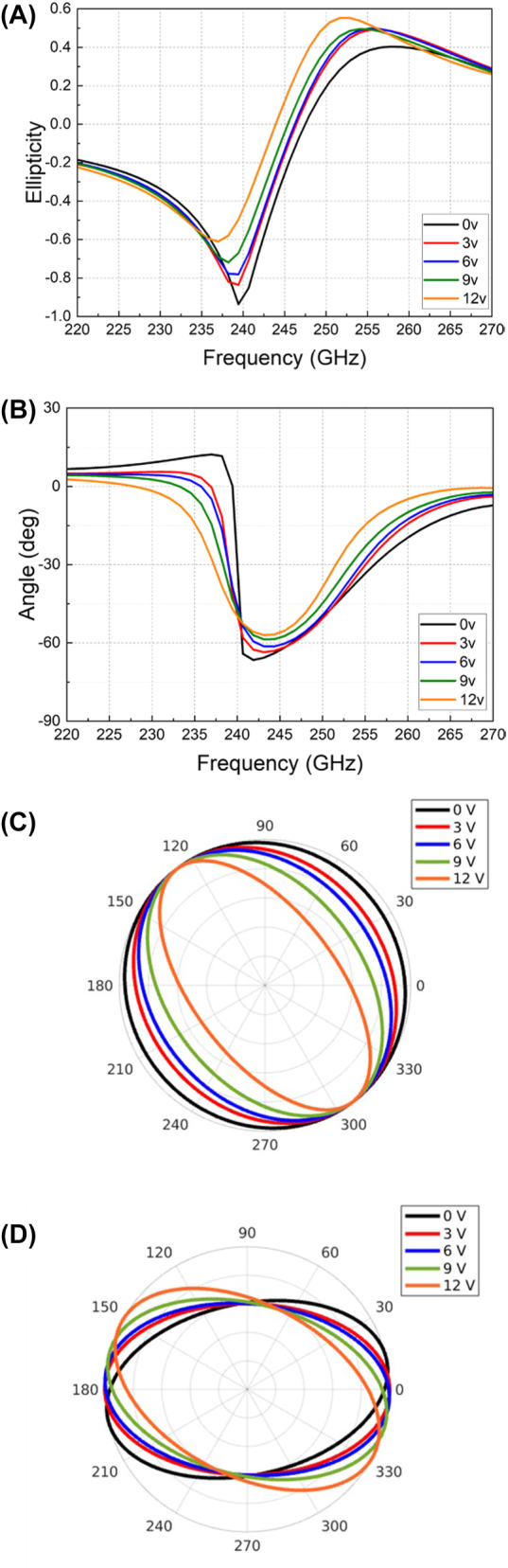
Measurement results. (A) Ellipticity. (B) Rotation angle. (C) Polarization variation in polar plot at 240 GHz. (D) Polarization variation in polar plot at 236 GHz.

The measured results of amplitudes and phase differences for the reflected *x*- and *y*-polarizations have been acquired by applying different biasing voltages and are plotted in Figure 8(A) and (B), respectively. Noticeable amplitude variations have been obtained, enabling a dynamic tuning of PCR from 0.67 to 0.77 at 245 GHz as shown in Figure 8(C). Compared to the simulation results in Figure 4(C), the PCR variation range from measurement is smaller than the simulation. This is owing to the performance deviation of the practically implemented graphene film from the ideally simulated one and the limited conductivity tuning range of the CVD graphene film used. To improve the practical performance, potentially, higher-quality and more uniform graphene films should be used once available. Meanwhile, with both variations of amplitudes and phases of the two orthogonal components, the electrically tuned polarization ellipticity and angle have been obtained, as demonstrated in Figure 9(A) and (B). At near 240 GHz, the ellipticity has been changed from −0.94 to −0.5 as the biasing voltage changes from 0 V to 12 V, showing the same polarization angle of −53°. At around 236 GHz, the reflected polarizations have different angles varied between 12° and −23° with a fixed ellipticity of −0.6. To visualize the tunability, the polar plots of the reflected polarization at these two frequencies are shown in Figure 9(C) and (D). Particularly, when the external biasing voltage is 0 V, the amplitudes of *Ex* and *Ey* at 240 GHz intersect at the same value of 0.5 (in Figure 8(A)), with a phase difference of near 90° (in Figure 8(B)), thereby producing a left-handed circular polarization with an ellipticity of near −1 (in Figure 9(A) and (C)). As the voltage varies, in Figure 8(A) and (B), the amplitude shows small variations at 240 GHz, while the phase difference has increased from 90° to 130°. Accordingly, the circular polarization has been changed to elliptical ones with different ellipticities. If the phase difference between *x*- and *y*-polarizations can be changed from 90° to 180° at a fixed frequency, a transition from a quarter-wave plate to a half-wave plate will be obtained. Due to the limited tunability of the applied graphene film in this experiment, the current phase tuning range only reaches 40° at a fixed frequency. Potentially, using higher-quality graphene film with a wider tunability may expand this range further.

As listed in Table 1, compared with the graphene-integrated polarization converters in Refs. [34], [35], this work features significantly smaller biasing voltages to achieve comparable or larger tuning ranges of the polarization state. Meanwhile, in contrast to Refs. [36], [37], no extra ion gel layer is required in this work for extending the device tunability within a small biasing range, thus simplifying the fabrication process and reducing the overall profile.

**Table 1:** Comparisons with other THz graphene-integrated polarization converters.

Ref.	Freq.	Tuneable component	Applied voltage	Tuning ellipticity	Tuning rotation angle
[34]	1.75 THz	Graphene	−125–25 V	–	20–40°
[35]	2.15 THz	Graphene	−35–100 V	−0.98 to −0.55	−21.5 to −12.5°
[36]	1.42 THz	Graphene + ion gel	0–4.8 V	–	30–40°
[37]	1.25 THz	Graphene + ion gel	0–2 V	0.6–1	–
This work	240 GHz	Graphene	0–12 V	−0.94 to −0.5	−23–12°

**Figure 10:**
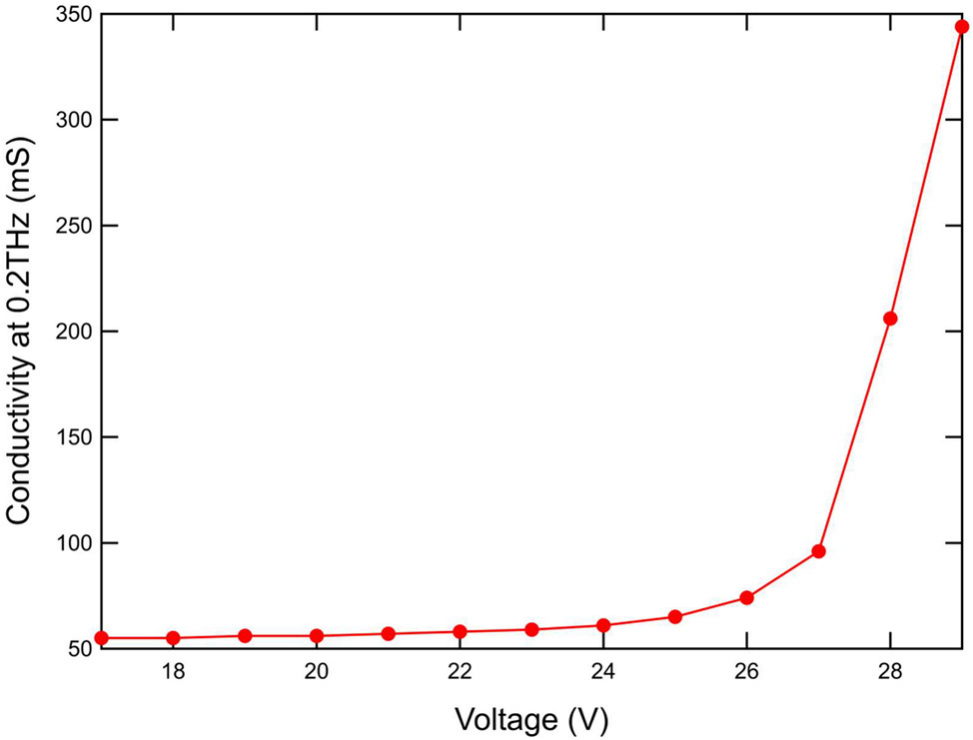
Measured conductivity of the graphene film versus voltage at 200 GHz.

Characterisation of the graphene conductivity was performed following the process by Bøggild et al. [40]. Figure 10 shows the measured conductivity versus biasing voltage for an unpatterned graphene film at 200 GHz. Before the graphene film breaks down, a conductivity tuning range of 50–350 mS is achieved. This informed our modelled conductivity range in the device simulation. The offset between the device biasing voltage (0–12 V) and the film characterisation one (17–29 V) is due to differing contact geometries between the bilayer patterned model and the characterisation sample. Since we cannot directly measure the conductivity of the patterned graphene film on the bilayer metasurface, Figure 10 gives an indication of the total tuning range available for the graphene film before breaking down, which is consistent with the modelling and device experimental data.

## 3 Conclusions

A graphene-enabled reconfigurable metasurface is developed for dynamic control of THz polarization state in terms of ellipticity and angle. A graphene-gold bilayer meta-structure is implemented, exhibiting broad electrical modulation depths, based on graphene’s light matter interactions within a small biasing range. By changing the biasing voltage from 0 V to 12 V, the incident linearly polarized wave has been converted into circularly and elliptically polarized waves with differing ellipticity and angle. The measurement results show a tuning range of 0.44 for polarization ellipticity with a fixed polarization angle at around 240 GHz, and a tuning range of 35° for polarization angle with a fixed ellipticity at 236 GHz. These ranges are comparable with state-of-the-art THz graphene-integrated metasurfaces, but achieved with much smaller biasing voltages without adding extra ion gel layers. This device holds great potentials in advanced sub-THz communication systems for data transmissions with multiple polarization states, as well as sensing applications such as chiral material and biomolecule characterizations.

## References

[1] Tonouchi M. (2007). Cutting-edge terahertz technology. *Nat. Photonics*.

[2] Song H.-J., Nagatsuma T. (2011). Present and future of terahertz communications. *IEEE Trans. Terahertz Sci. Technol.*.

[3] Federici J. F. (2005). THz imaging and sensing for security applications – explosives, weapons and drugs. *Semicond. Sci. Technol.*.

[4] Oshima N., Hashimoto K., Suzuki S., Asada M. (2017). Terahertz wireless data transmission with frequency and polarization division multiplexing using resonant-tunneling-diode oscillators. *IEEE Trans. Terahertz Sci. Technol.*.

[5] Zhang L., Zhong H., Deng C., Zhang C., Zhao Y. (2010). Characterization of birefringent material using polarization-controlled terahertz spectroscopy. *Opt. Express*.

[6] Xu J. (2004). Terahertz circular dichroism spectroscopy of biomolecules. *Proc. SPIE*.

[7] Valev V. K., Baumberg J. J., Sibilia C., Verbiest T. (2013). Chirality and chiroptical effects in plasmonic nanostructures: fundamentals, recent progress, and outlook. *Adv. Mater.*.

[8] Hibberd M. T., Lake D. S., Johansson N. A. B., Thomson T., Jamison S. P., Graham D. M. (2019). Magnetic-field tailoring of the terahertz polarization emitted from a spintronic source. *Appl. Phys. Lett.*.

[9] Berry M. V., Dennis M. R. (2003). The optical singularities of birefringent dichroic chiral crystals. *Proc. R. Soc. Lond. Ser. A Math. Phys. Eng. Sci.*.

[10] Hirota Y., Hattori R., Tani M., Hangyo M. (2006). Polarization modulation of terahertz electromagnetic radiation by four-contact photoconductive antenna. *Opt. Express*.

[11] Cong L., Pitchappa P., Wang N., Singh R. (2019). Electrically programmable terahertz diatomic metamolecules for chiral optical control. *Research*.

[12] Holloway C. L., Kuester E. F., Gordon J. A., O’Hara J., Booth J., Smith D. R. (2012). An overview of the theory and applications of metasurfaces: the two-dimensional equivalents of metamaterials. *IEEE Antenn. Propag. Mag.*.

[13] Nicholls L. H. (2017). Ultrafast synthesis and switching of light polarization in nonlinear anisotropic metamaterials. *Nat. Photonics*.

[14] Yu P., Li J., Liu N. (2021). Electrically tunable optical metasurfaces for dynamic polarization conversion. *Nano Lett*..

[15] Biswas S., Grajower M. Y., Watanabe K., Taniguchi T., Atwater H. A. (2021). Broadband electro-optic polarization conversion with atomically thin black phosphorus. *Science*.

[16] Meng C., Thrane P. C. V., Ding F., Bozhevolnyi S. I. (2022). Full-range birefringence control with piezoelectric MEMS-based metasurfaces. *Nat. Commun.*.

[17] Venkatesh S., Lu X., Saeidi H., Sengupta K. (2022). A programmable terahertz metasurface with circuit-coupled meta-elements in silicon chips: creating low-cost, large-scale, reconfigurable terahertz metasurfaces. *IEEE Antenn. Propag. Mag.*.

[18] Fu X., Yang F., Liu C., Wu X., Cui T. J. (2020). Terahertz beam steering technologies: from phased arrays to field-programmable metasurfaces. *Adv. Opt. Mater.*.

[19] Perez-Palomino G. (2015). Design and demonstration of an electronically scanned reflectarray antenna at 100 GHz using multiresonant cells based on liquid crystals. *IEEE Trans. Antenn. Propag.*.

[20] Kim H. T. (2005). Raman study of electric-field-induced first-order metal-insulator transition in VO2-based devices. *Appl. Phys. Lett.*.

[21] Squires A. D. (2022). Electrically tuneable terahertz metasurface enabled by a graphene/gold bilayer structure. *Commun. Mater.*.

[22] He X. (2015). Tunable terahertz graphene metamaterials. *Carbon*.

[23] Zeng F., Ye L., Li L., Wang Z., Zhao W., Zhang Y. (2019). Tunable mid-infrared dual-band and broadband cross-polarization converters based on U-shaped graphene metamaterials. *Opt. Express*.

[24] Yuan K., Ye L. (2023). Design and simulation of high-efficiency broadband terahertz graphene composite waveguide modulators. *IEEE J. Sel. Top. Quantum Electron.*.

[25] Ye L., Yuan K., Zhu C., Zhang Y., Zhang Y., Lai K. (2021). Broadband high-efficiency near-infrared graphene phase modulators enabled by metal–nanoribbon integrated hybrid plasmonic waveguides. *Nanophotonics*.

[26] Yuan X., Chen J., Wu J., Yan X., Zhang Y., Zhang X. (2022). Graphene-based tunable linear and linear-to-circular polarization converters in the THz band. *Results Phys.*.

[27] Quader S., Zhang J., Akram M. R., Zhu W. (2020). Graphene-based high-efficiency broadband tunable linear-to-circular polarization converter for terahertz waves. *IEEE J. Sel. Top. Quantum Electron.*.

[28] Zhang H. (2023). Tunable broadband transmissive terahertz cross-polarization converter enabled by a hybrid metal-graphene metasurface. *Results Phys.*.

[29] Amin M., Siddiqui O., Farhat M. (2020). Linear and circular dichroism in graphene-based reflectors for polarization control. *Phys. Rev. Appl.*.

[30] Cheng Y., Wang J. (2021). Tunable terahertz circular polarization convertor based on graphene metamaterial. *Diam. Relat. Mater.*.

[31] Ghosh S. K., Das S., Bhattacharyya S. (2022). Terahertz wave conversion from linear to circular polarization by graphene metasurface featuring ultrawideband tunability. *J. Light.*.

[32] Cheng Y., Zhu X., Li J., Fu C., Luo H., Wu L. (2021). Terahertz broadband tunable reflective cross-polarization convertor based on complementary cross-shaped graphene metasurface. *Phys. E Low-dimens. Syst. Nanostruct.*.

[33] Barkabian M., Sharifi N., Granpayeh N. (2021). Multi-functional high-efficiency reflective polarization converter based on an ultra-thin graphene metasurface in the THz band. *Opt. Express*.

[34] Kindness S. J. (2019). Graphene-integrated metamaterial device for all-electrical polarization control of terahertz quantum cascade lasers. *ACS Photonics*.

[35] Kindness S. J. (2020). A terahertz chiral metamaterial modulator. *Adv. Opt. Mater.*.

[36] Kim T.-T. (2017). Electrical access to critical coupling of circularly polarized waves in graphene chiral metamaterials. *Sci. Adv.*.

[37] Park H.-W. (2023). Electrically tunable THz graphene metasurface wave retarders. *Nanophotonics*.

[38] Chen Z. (2022). Broadband graphene-based electro-optic chiral polarization conversion for terahertz pulse shaping. *ACS Photonics*.

[39] Seo D. H. (2017). Single-step ambient-air synthesis of graphene from renewable precursors as electrochemical genosensor. *Nat. Commun.*.

[40] Bøggild P. (2017). Mapping the electrical properties of large-area graphene. *2D Mater.*.

